# Crystal structure of (5′*S*,8′*S*)-3-(2,5-di­methyl­phen­yl)-8-meth­oxy-3-nitro-1-aza­spiro­[4.5]decane-2,4-dione

**DOI:** 10.1107/S2056989015004715

**Published:** 2015-03-14

**Authors:** Gao-Bo Hu, Da-Wei Jiang, Jiang-Yan Li, Yan Rao, Li-Yuan Jiang

**Affiliations:** a Medical College, Quzhou College of Technology, Quzhou 324000, People’s Republic of China

**Keywords:** crystal structure, 1-aza­spiro­[4.5]decane-2,4-dione, hydrogen bonding, pesticide, spiro­tetra­mat

## Abstract

The title compound, C_18_H_22_N_2_O_5_, was synthesized by nitrification of its enol precursor. The pyrrolidine ring plane adopts a twisted conformation about the C—C bond linking the spiro centre and the C=O group remote from the N atom. It makes dihedral angles of 71.69 (9) and 88.92 (9)°, respectively, with the benzene ring plane and the plane defined by the four C atoms that form the seat of the of the cyclo­hexane chair. At the spiro centre, the NH group is axial and the C=O group is equatorial with respect to the cyclo­hexane ring. In the crystal, inversion dimers linked by pairs of N—H⋯O hydrogen bonds generate *R*_2_^2^(8) loops. The dimers are linked by C—H⋯O inter­actions, generating a three-dimensional network.

## Related literature   

For the pesticide spiro­tetra­mat, the central unit of the title compound, see: Fischer & Weiss (2008 ▸); Maus (2008 ▸); Bruck *et al.* (2009 ▸); Campbell *et al.* (1985 ▸); Schobert & Schlenk (2008 ▸). For structures of spiro­tetra­mat derivatives, see: Fischer *et al.* (2010 ▸).
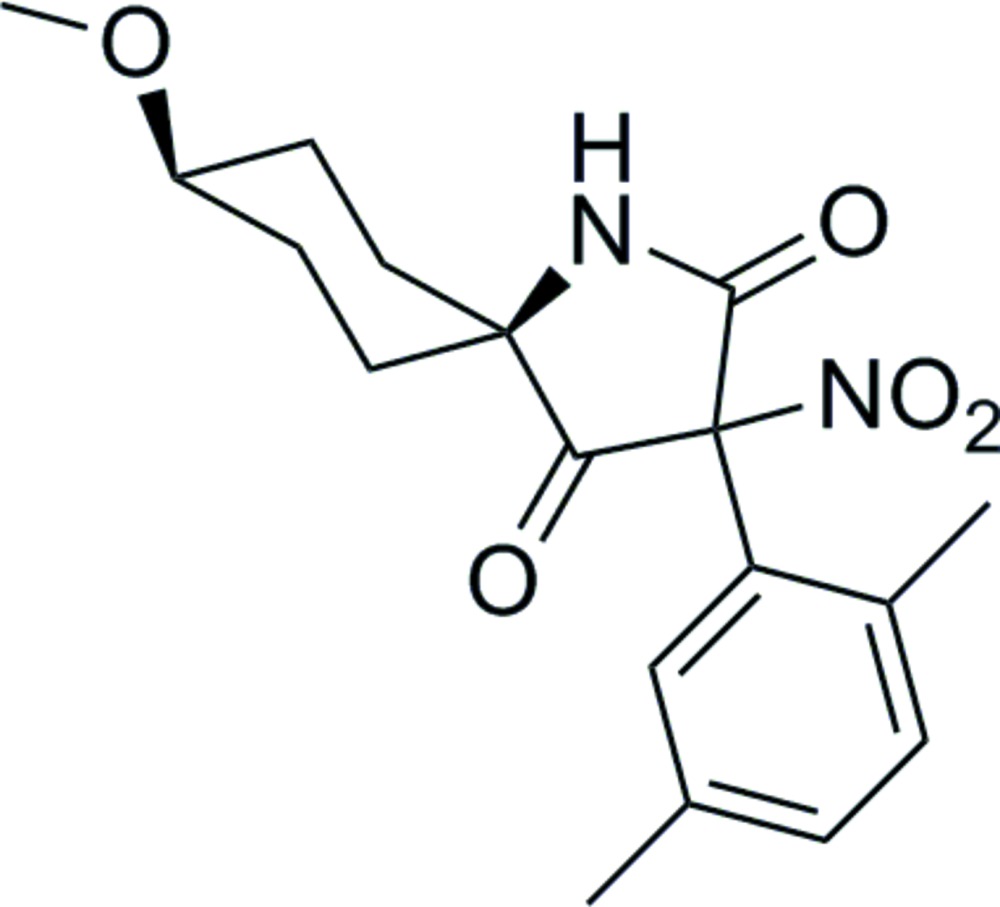


## Experimental   

### Crystal data  

C_18_H_22_N_2_O_5_
*M* *_r_* = 346.38
Monoclinic, 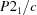

*a* = 9.5707 (9) Å
*b* = 8.4181 (7) Å
*c* = 22.8720 (19) Å
β = 100.703 (8)°
*V* = 1810.7 (3) Å^3^
*Z* = 4
Mo *K*α radiation
μ = 0.09 mm^−1^
*T* = 170 K
0.36 × 0.32 × 0.23 mm

### Data collection  

Agilent Xcalibur (Atlas, Gemini ultra) diffractometer
Absorption correction: multi-scan (*CrysAlis PRO*; Agilent, 2011) *T*_min_ = 0.954, *T*_max_ = 1.000
6891 measured reflections
3308 independent reflections
2600 reflections with *I* > 2σ(*I*)
*R*_int_ = 0.034

### Refinement  

*R*[*F* ^2^ > 2σ(*F* ^2^)] = 0.050
*wR*(*F* ^2^) = 0.139
*S* = 1.04
3308 reflections
229 parameters
H-atom parameters constrained
Δρ_max_ = 0.60 e Å^−3^
Δρ_min_ = −0.31 e Å^−3^

### 

Data collection: *CrysAlis PRO* (Agilent, 2011); cell refinement: *CrysAlis PRO*; data reduction: *CrysAlis PRO*; program(s) used to solve structure: *SHELXS97* (Sheldrick, 2008 ▸); program(s) used to refine structure: *SHELXL97* (Sheldrick, 2008 ▸); molecular graphics: *OLEX2* (Dolomanov *et al.*, 2009 ▸); software used to prepare material for publication: *OLEX2*.

## Supplementary Material

Crystal structure: contains datablock(s) I. DOI: 10.1107/S2056989015004715/hb7342sup1.cif

Structure factors: contains datablock(s) I. DOI: 10.1107/S2056989015004715/hb7342Isup2.hkl

Supporting information file. DOI: 10.1107/S2056989015004715/hb7342Isup3.cml

**Fig. 1.**
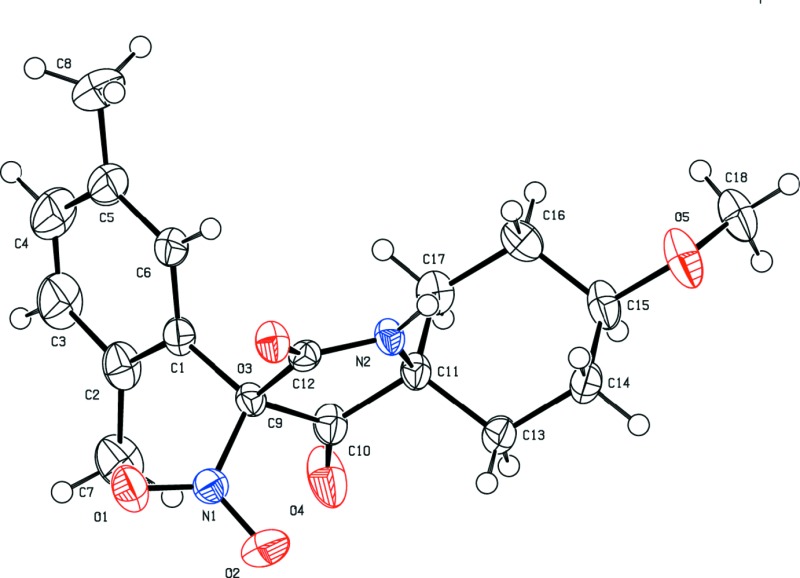
The molecular structure of title molecule, showing 50% displacement ellipsoids.

. DOI: 10.1107/S2056989015004715/hb7342fig1.tif
The mol­ecular structure of title mol­ecule, showing 50% displacement ellipsoids.

**Fig. 2.**
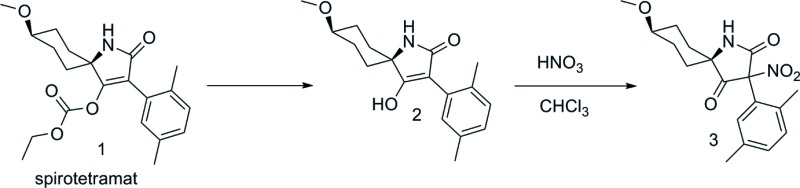
Reaction scheme.

. DOI: 10.1107/S2056989015004715/hb7342fig2.tif
Reaction scheme.

CCDC reference: 1052631

Additional supporting information:  crystallographic information; 3D view; checkCIF report

## Figures and Tables

**Table 1:** Hydrogen-bond geometry (, )

*D*H*A*	*D*H	H*A*	*D* *A*	*D*H*A*
N2H2O3^i^	0.88	2.02	2.8853(19)	167
C4H4O5^ii^	0.95	2.57	3.287(3)	132
C7H7*B*O1^iii^	0.98	2.49	3.454(3)	168
C14H14*B*O2^iv^	0.99	2.54	3.265(3)	130

Symmetry codes: (i) 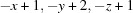; (ii) 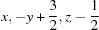; (iii) 
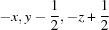
; (iv) 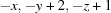.

## supplementary crystallographic information

### S1. Comment

Spirotetramat is a new systemic insecticide which chemically belongs to the class of spirocyclic tetramic acid derivatives and be developed by Bayer CropScience AG (Fischer *et al.*, 2008; Maus, 2008). A unique mode of action coupled with a high degree of activity on targeted pests and low toxicity to nontarget organisms make spirocyclic tetronic acid compounds as a new tool for integrated pest management (Bruck *et al.*, 2009; Campbell *et al.*,1985; Schobert *et al.*, 2008) In order to study the influence of new substituents on the activity of the Spirotetramat derivative, the title compound, has been synthesized and its structure has been determined (Fig. 1). The molecule contains one benzene ring, one six membered ring, and one five membered ring. The cyclohexane ring adopts a chair conformation;the C13, C14, C16 and C17 atoms are on one plane with C15 and C11 deviating by 0.658 (5)and -0.676 (9) Å, respectively. There are three planes in the molecule: atoms of C10, C11, C12 and N2 generate the pyrrolidine plane (I), C1—C6 yield the benzene plane (II) and C13—C14 and C16—C17 form the cyclohexane plane(III). The angle between planes I and II is 71.69 (9) °, and that between planes I and III is 88.92 (9) °. The space arrangement might result from the space factor between groups.

### S2. Experimental

A solution of fuming nitric acid (0.92 g, 16mmoL) in anhydrous chloroform (10 ml) was added dropwise to a solution of compound 2 (1.78 g, 5.9mmoL) in anhydrous chloroform (20 ml) at 0 degree and stirred for 2 h. The reaction mixture was then washed with ice water (15 ml), and saturated sodium chloride solution and dried over anhydrous Na_2_SO_4_. The solvent was evaporated, and the residual solid was crystallized from ethanol to afford 1.84 g compound 3 as a pale yellow solid: yield 90%. The 1H NMR, 13 C-NMR and ESI-MS data testified the title compound's structure. ESI-MS: 347 (*M*+H)+ (100%); 1H NMR (500 MHz, CDCl3): 7.48 (s, 1H, –NH–),7.55 (s, 1H, Ph—H), 7.14 (d, 1H, Ph—H), 7.07 (d, 1H, Ph—H), 3.54 (s, 3H, –OCH3), 3.34–3.33 (m, 1H, –CH—O—C–), 2.36 (s, 3H, Ph—Me), 2.27 (s, 3H, Ph—Me), 2.12–2.07 (m, 4H, Cyclohexane-H4), 1.99–1.56 (m, 4H, Cyclohexane-H4); 13 C-NMR (100 MHz, CDCl3): 199.9, 165.1, 136.7, 136.0, 133.2, 131.5, 129.7, 127.4, 95.8, 76.8, 75.6, 66.1, 55.7, 32.5, 31.5, 26.4, 26.3, 21.0, 20.3.

### S3. Refinement

The H atoms were geometrically placed (C–H = 0.93–0.98 Å) and refined as riding with *U*_iso_(H) = 1.2*U*_eq_(C).

### Crystal data

**Table:**

C_18_H_22_N_2_O_5_	*F*(000) = 736
*M**_r_* = 346.38	*D*_x_ = 1.271 Mg m^−^^3^
Monoclinic, *P*2_1_/*c*	Mo *K*α radiation, λ = 0.71073 Å
*a* = 9.5707 (9) Å	Cell parameters from 2205 reflections
*b* = 8.4181 (7) Å	θ = 3.2–29.5°
*c* = 22.8720 (19) Å	µ = 0.09 mm^−^^1^
β = 100.703 (8)°	*T* = 170 K
*V* = 1810.7 (3) Å^3^	Block, colourless
*Z* = 4	0.36 × 0.32 × 0.23 mm

### Data collection

**Table:**

Agilent Xcalibur (Atlas, Gemini ultra) diffractometer	3308 independent reflections
Radiation source: Enhance (Mo) X-ray Source	2600 reflections with *I* > 2σ(*I*)
Graphite monochromator	*R*_int_ = 0.034
Detector resolution: 10.3592 pixels mm^-1^	θ_max_ = 25.4°, θ_min_ = 3.3°
ω scans	*h* = −11→7
Absorption correction: multi-scan (*CrysAlis PRO*; Agilent, 2011)	*k* = −8→10
*T*_min_ = 0.954, *T*_max_ = 1.000	*l* = −22→27
6891 measured reflections	

### Refinement

**Table:**

Refinement on *F*^2^	Primary atom site location: structure-invariant direct methods
Least-squares matrix: full	Secondary atom site location: difference Fourier map
*R*[*F*^2^ > 2σ(*F*^2^)] = 0.050	Hydrogen site location: inferred from neighbouring sites
*wR*(*F*^2^) = 0.139	H-atom parameters constrained
*S* = 1.04	*w* = 1/[σ^2^(*F*_o_^2^) + (0.0602*P*)^2^ + 0.8521*P*] where *P* = (*F*_o_^2^ + 2*F*_c_^2^)/3
3308 reflections	(Δ/σ)_max_ < 0.001
229 parameters	Δρ_max_ = 0.60 e Å^−^^3^
0 restraints	Δρ_min_ = −0.31 e Å^−^^3^

### Special details

**Table:**

Experimental. Absorption correction: CrysAlisPro, Agilent Technologies, Version 1.171.35.11 (release 16-05-2011 CrysAlis171 .NET) (compiled May 16 2011,17:55:39) Empirical absorption correction using spherical harmonics, implemented in SCALE3 ABSPACK scaling algorithm.
Geometry. All e.s.d.'s (except the e.s.d. in the dihedral angle between two l.s. planes) are estimated using the full covariance matrix. The cell e.s.d.'s are taken into account individually in the estimation of e.s.d.'s in distances, angles and torsion angles; correlations between e.s.d.'s in cell parameters are only used when they are defined by crystal symmetry. An approximate (isotropic) treatment of cell e.s.d.'s is used for estimating e.s.d.'s involving l.s. planes.
Refinement. Refinement of *F*^2^ against ALL reflections. The weighted *R*-factor *wR* and goodness of fit *S* are based on *F*^2^, conventional *R*-factors *R* are based on *F*, with *F* set to zero for negative *F*^2^. The threshold expression of *F*^2^ > σ(*F*^2^) is used only for calculating *R*-factors(gt) *etc*. and is not relevant to the choice of reflections for refinement. *R*-factors based on *F*^2^ are statistically about twice as large as those based on *F*, and *R*- factors based on ALL data will be even larger.

### Fractional atomic coordinates and isotropic or equivalent isotropic displacement parameters (Å^2^)

**Table:**

	*x*	*y*	*z*	*U*_iso_*/*U*_eq_	
O1	0.18294 (17)	1.31285 (17)	0.34437 (6)	0.0406 (4)	
O2	0.09810 (17)	1.2327 (2)	0.42014 (7)	0.0500 (5)	
O3	0.44250 (14)	1.14145 (16)	0.43813 (5)	0.0295 (3)	
O4	−0.00240 (16)	0.9051 (2)	0.37430 (8)	0.0579 (5)	
O5	0.32239 (19)	0.4533 (2)	0.56929 (7)	0.0558 (5)	
N1	0.16126 (17)	1.2114 (2)	0.37880 (7)	0.0281 (4)	
N2	0.33749 (16)	0.90936 (19)	0.45886 (6)	0.0245 (4)	
H2	0.4057	0.8790	0.4880	0.029*	
C1	0.2650 (2)	1.0141 (2)	0.31529 (8)	0.0277 (4)	
C2	0.1642 (2)	1.0202 (3)	0.26206 (9)	0.0381 (5)	
C3	0.2143 (3)	0.9836 (3)	0.21029 (10)	0.0558 (7)	
H3	0.1491	0.9859	0.1735	0.067*	
C4	0.3526 (3)	0.9447 (3)	0.20978 (11)	0.0612 (8)	
H4	0.3801	0.9187	0.1731	0.073*	
C5	0.4534 (3)	0.9422 (3)	0.26167 (10)	0.0483 (6)	
C6	0.4058 (2)	0.9761 (2)	0.31421 (9)	0.0342 (5)	
H6	0.4721	0.9732	0.3507	0.041*	
C7	0.0114 (3)	1.0702 (3)	0.25703 (10)	0.0509 (7)	
H7A	−0.0125	1.0761	0.2968	0.076*	
H7B	−0.0505	0.9924	0.2330	0.076*	
H7C	−0.0024	1.1747	0.2379	0.076*	
C8	0.6086 (3)	0.9084 (4)	0.26190 (13)	0.0732 (9)	
H8A	0.6263	0.7941	0.2671	0.110*	
H8B	0.6674	0.9663	0.2947	0.110*	
H8C	0.6327	0.9428	0.2241	0.110*	
C9	0.22283 (19)	1.0470 (2)	0.37501 (8)	0.0237 (4)	
C10	0.1216 (2)	0.9211 (2)	0.39383 (8)	0.0305 (5)	
C11	0.20633 (19)	0.8179 (2)	0.44258 (8)	0.0258 (4)	
C12	0.34921 (19)	1.0401 (2)	0.42782 (8)	0.0228 (4)	
C13	0.1293 (2)	0.8034 (3)	0.49537 (9)	0.0346 (5)	
H13A	0.1177	0.9104	0.5118	0.042*	
H13B	0.0334	0.7582	0.4815	0.042*	
C14	0.2111 (2)	0.6980 (3)	0.54417 (9)	0.0374 (5)	
H14A	0.3025	0.7494	0.5613	0.045*	
H14B	0.1555	0.6857	0.5763	0.045*	
C15	0.2394 (2)	0.5373 (3)	0.52057 (9)	0.0384 (5)	
H15	0.1470	0.4806	0.5074	0.046*	
C16	0.3164 (2)	0.5505 (2)	0.46846 (9)	0.0349 (5)	
H16A	0.3296	0.4430	0.4527	0.042*	
H16B	0.4117	0.5974	0.4823	0.042*	
C17	0.2336 (2)	0.6532 (2)	0.41893 (9)	0.0328 (5)	
H17A	0.1417	0.6015	0.4026	0.039*	
H17B	0.2882	0.6635	0.3864	0.039*	
C18	0.3060 (3)	0.2894 (3)	0.56796 (12)	0.0524 (7)	
H18A	0.3302	0.2475	0.5311	0.079*	
H18B	0.2071	0.2627	0.5696	0.079*	
H18C	0.3691	0.2424	0.6023	0.079*	

### Atomic displacement parameters (Å^2^)

**Table:**

	*U*^11^	*U*^22^	*U*^33^	*U*^12^	*U*^13^	*U*^23^
O1	0.0575 (10)	0.0297 (8)	0.0332 (8)	0.0042 (7)	0.0051 (7)	0.0052 (7)
O2	0.0483 (10)	0.0597 (11)	0.0476 (9)	0.0133 (8)	0.0233 (8)	−0.0020 (8)
O3	0.0273 (7)	0.0311 (7)	0.0276 (7)	−0.0056 (6)	−0.0009 (5)	0.0041 (6)
O4	0.0303 (9)	0.0716 (12)	0.0634 (11)	−0.0161 (8)	−0.0136 (8)	0.0371 (10)
O5	0.0640 (12)	0.0480 (10)	0.0476 (10)	−0.0009 (8)	−0.0099 (8)	0.0225 (8)
N1	0.0251 (9)	0.0339 (10)	0.0245 (8)	0.0026 (7)	0.0021 (7)	0.0004 (8)
N2	0.0227 (8)	0.0274 (9)	0.0214 (8)	−0.0010 (7)	−0.0009 (6)	0.0055 (7)
C1	0.0367 (11)	0.0236 (10)	0.0228 (9)	−0.0002 (8)	0.0052 (8)	0.0010 (8)
C2	0.0505 (14)	0.0352 (12)	0.0253 (10)	−0.0028 (10)	−0.0015 (9)	−0.0014 (9)
C3	0.084 (2)	0.0578 (16)	0.0226 (11)	0.0047 (15)	0.0014 (11)	−0.0071 (11)
C4	0.093 (2)	0.0641 (18)	0.0323 (13)	0.0112 (16)	0.0262 (14)	−0.0061 (12)
C5	0.0671 (17)	0.0443 (14)	0.0393 (13)	0.0146 (12)	0.0247 (12)	0.0037 (11)
C6	0.0424 (12)	0.0349 (11)	0.0267 (10)	0.0065 (10)	0.0106 (9)	0.0048 (9)
C7	0.0493 (15)	0.0596 (16)	0.0350 (12)	−0.0003 (12)	−0.0145 (10)	−0.0009 (12)
C8	0.076 (2)	0.089 (2)	0.0660 (18)	0.0305 (18)	0.0426 (16)	0.0096 (17)
C9	0.0233 (10)	0.0254 (10)	0.0212 (9)	0.0009 (8)	0.0009 (7)	0.0036 (8)
C10	0.0258 (11)	0.0363 (11)	0.0275 (10)	−0.0049 (9)	0.0003 (8)	0.0060 (9)
C11	0.0215 (10)	0.0308 (10)	0.0237 (9)	−0.0033 (8)	0.0011 (7)	0.0057 (8)
C12	0.0207 (9)	0.0286 (10)	0.0192 (9)	0.0010 (8)	0.0039 (7)	−0.0007 (8)
C13	0.0297 (11)	0.0420 (12)	0.0340 (11)	0.0025 (9)	0.0106 (9)	0.0106 (10)
C14	0.0338 (12)	0.0508 (14)	0.0294 (11)	0.0028 (10)	0.0108 (9)	0.0135 (10)
C15	0.0332 (12)	0.0409 (13)	0.0367 (11)	−0.0065 (10)	−0.0047 (9)	0.0161 (10)
C16	0.0354 (12)	0.0279 (11)	0.0381 (12)	0.0004 (9)	−0.0021 (9)	−0.0013 (9)
C17	0.0348 (12)	0.0326 (11)	0.0289 (10)	−0.0060 (9)	0.0000 (8)	−0.0008 (9)
C18	0.0645 (17)	0.0363 (13)	0.0544 (15)	0.0038 (12)	0.0059 (12)	0.0190 (12)

### Geometric parameters (Å, º)

**Table:**

O1—N1	1.206 (2)	C8—H8A	0.9800
O2—N1	1.226 (2)	C8—H8B	0.9800
O3—C12	1.226 (2)	C8—H8C	0.9800
O4—C10	1.195 (2)	C9—C10	1.550 (3)
O5—C15	1.430 (2)	C9—C12	1.544 (2)
O5—C18	1.388 (3)	C10—C11	1.523 (3)
N1—C9	1.513 (2)	C11—C13	1.532 (3)
N2—H2	0.8800	C11—C17	1.529 (3)
N2—C11	1.461 (2)	C13—H13A	0.9900
N2—C12	1.325 (2)	C13—H13B	0.9900
C1—C2	1.407 (3)	C13—C14	1.523 (3)
C1—C6	1.390 (3)	C14—H14A	0.9900
C1—C9	1.520 (3)	C14—H14B	0.9900
C2—C3	1.392 (3)	C14—C15	1.500 (3)
C2—C7	1.506 (3)	C15—H15	1.0000
C3—H3	0.9500	C15—C16	1.518 (3)
C3—C4	1.366 (4)	C16—H16A	0.9900
C4—H4	0.9500	C16—H16B	0.9900
C4—C5	1.384 (4)	C16—C17	1.525 (3)
C5—C6	1.391 (3)	C17—H17A	0.9900
C5—C8	1.511 (4)	C17—H17B	0.9900
C6—H6	0.9500	C18—H18A	0.9800
C7—H7A	0.9800	C18—H18B	0.9800
C7—H7B	0.9800	C18—H18C	0.9800
C7—H7C	0.9800		
			
C18—O5—C15	115.49 (19)	N2—C11—C10	101.63 (15)
O1—N1—O2	124.68 (18)	N2—C11—C13	110.96 (15)
O1—N1—C9	119.64 (15)	N2—C11—C17	111.93 (15)
O2—N1—C9	115.55 (16)	C10—C11—C13	110.73 (16)
C11—N2—H2	121.5	C10—C11—C17	111.17 (15)
C12—N2—H2	121.5	C17—C11—C13	110.18 (16)
C12—N2—C11	117.08 (15)	O3—C12—N2	127.30 (17)
C2—C1—C9	121.12 (18)	O3—C12—C9	124.13 (16)
C6—C1—C2	120.31 (18)	N2—C12—C9	108.57 (15)
C6—C1—C9	118.57 (17)	C11—C13—H13A	109.3
C1—C2—C7	125.23 (19)	C11—C13—H13B	109.3
C3—C2—C1	116.0 (2)	H13A—C13—H13B	108.0
C3—C2—C7	118.7 (2)	C14—C13—C11	111.56 (16)
C2—C3—H3	118.4	C14—C13—H13A	109.3
C4—C3—C2	123.2 (2)	C14—C13—H13B	109.3
C4—C3—H3	118.4	C13—C14—H14A	109.4
C3—C4—H4	119.4	C13—C14—H14B	109.4
C3—C4—C5	121.2 (2)	H14A—C14—H14B	108.0
C5—C4—H4	119.4	C15—C14—C13	111.35 (17)
C4—C5—C6	116.8 (2)	C15—C14—H14A	109.4
C4—C5—C8	122.3 (2)	C15—C14—H14B	109.4
C6—C5—C8	121.0 (2)	O5—C15—C14	106.08 (17)
C1—C6—C5	122.4 (2)	O5—C15—H15	109.3
C1—C6—H6	118.8	O5—C15—C16	111.55 (18)
C5—C6—H6	118.8	C14—C15—H15	109.3
C2—C7—H7A	109.5	C14—C15—C16	111.32 (17)
C2—C7—H7B	109.5	C16—C15—H15	109.3
C2—C7—H7C	109.5	C15—C16—H16A	109.4
H7A—C7—H7B	109.5	C15—C16—H16B	109.4
H7A—C7—H7C	109.5	C15—C16—C17	111.29 (18)
H7B—C7—H7C	109.5	H16A—C16—H16B	108.0
C5—C8—H8A	109.5	C17—C16—H16A	109.4
C5—C8—H8B	109.5	C17—C16—H16B	109.4
C5—C8—H8C	109.5	C11—C17—H17A	109.5
H8A—C8—H8B	109.5	C11—C17—H17B	109.5
H8A—C8—H8C	109.5	C16—C17—C11	110.53 (16)
H8B—C8—H8C	109.5	C16—C17—H17A	109.5
N1—C9—C1	112.92 (15)	C16—C17—H17B	109.5
N1—C9—C10	109.82 (15)	H17A—C17—H17B	108.1
N1—C9—C12	104.28 (14)	O5—C18—H18A	109.5
C1—C9—C10	114.16 (16)	O5—C18—H18B	109.5
C1—C9—C12	113.32 (15)	O5—C18—H18C	109.5
C12—C9—C10	101.33 (14)	H18A—C18—H18B	109.5
O4—C10—C9	127.03 (18)	H18A—C18—H18C	109.5
O4—C10—C11	124.44 (18)	H18B—C18—H18C	109.5
C11—C10—C9	108.53 (15)		
			
O1—N1—C9—C1	−18.9 (2)	C6—C1—C9—N1	120.84 (19)
O1—N1—C9—C10	−147.60 (16)	C6—C1—C9—C10	−112.8 (2)
O1—N1—C9—C12	104.50 (18)	C6—C1—C9—C12	2.6 (2)
O2—N1—C9—C1	165.05 (16)	C7—C2—C3—C4	176.7 (3)
O2—N1—C9—C10	36.4 (2)	C8—C5—C6—C1	177.3 (2)
O2—N1—C9—C12	−71.51 (19)	C9—C1—C2—C3	−178.3 (2)
O4—C10—C11—N2	−167.1 (2)	C9—C1—C2—C7	4.9 (3)
O4—C10—C11—C13	−49.2 (3)	C9—C1—C6—C5	179.1 (2)
O4—C10—C11—C17	73.6 (3)	C9—C10—C11—N2	12.77 (19)
O5—C15—C16—C17	−174.73 (17)	C9—C10—C11—C13	130.69 (17)
N1—C9—C10—O4	53.2 (3)	C9—C10—C11—C17	−106.48 (18)
N1—C9—C10—C11	−126.63 (16)	C10—C9—C12—O3	−164.71 (18)
N1—C9—C12—O3	−50.6 (2)	C10—C9—C12—N2	14.88 (19)
N1—C9—C12—N2	128.95 (15)	C10—C11—C13—C14	178.93 (18)
N2—C11—C13—C14	−69.0 (2)	C10—C11—C17—C16	−179.12 (16)
N2—C11—C17—C16	68.0 (2)	C11—N2—C12—O3	171.67 (18)
C1—C2—C3—C4	−0.4 (4)	C11—N2—C12—C9	−7.9 (2)
C1—C9—C10—O4	−74.7 (3)	C11—C13—C14—C15	−55.5 (2)
C1—C9—C10—C11	105.40 (18)	C12—N2—C11—C10	−3.1 (2)
C1—C9—C12—O3	72.5 (2)	C12—N2—C11—C13	−120.87 (18)
C1—C9—C12—N2	−107.87 (17)	C12—N2—C11—C17	115.59 (18)
C2—C1—C6—C5	−0.3 (3)	C12—C9—C10—O4	163.1 (2)
C2—C1—C9—N1	−59.8 (2)	C12—C9—C10—C11	−16.77 (19)
C2—C1—C9—C10	66.6 (2)	C13—C11—C17—C16	−56.0 (2)
C2—C1—C9—C12	−178.06 (18)	C13—C14—C15—O5	176.97 (17)
C2—C3—C4—C5	−1.3 (4)	C13—C14—C15—C16	55.5 (2)
C3—C4—C5—C6	2.1 (4)	C14—C15—C16—C17	−56.5 (2)
C3—C4—C5—C8	−176.6 (3)	C15—C16—C17—C11	56.7 (2)
C4—C5—C6—C1	−1.3 (3)	C17—C11—C13—C14	55.5 (2)
C6—C1—C2—C3	1.1 (3)	C18—O5—C15—C14	150.9 (2)
C6—C1—C2—C7	−175.7 (2)	C18—O5—C15—C16	−87.7 (2)

### Hydrogen-bond geometry (Å, º)

**Table:**

*D*—H···*A*	*D*—H	H···*A*	*D*···*A*	*D*—H···*A*
N2—H2···O3^i^	0.88	2.02	2.8853 (19)	167
C4—H4···O5^ii^	0.95	2.57	3.287 (3)	132
C7—H7*B*···O1^iii^	0.98	2.49	3.454 (3)	168
C14—H14*B*···O2^iv^	0.99	2.54	3.265 (3)	130

Symmetry codes: (i) −*x*+1, −*y*+2, −*z*+1; (ii) *x*, −*y*+3/2, *z*−1/2; (iii) −*x*, *y*−1/2, −*z*+1/2; (iv) −*x*, −*y*+2, −*z*+1.
