# Supplementary figures and images for: Crystal structure of (5′S,8′S)-3-(2,5-di­methyl­phen­yl)-8-meth­oxy-3-nitro-1-aza­spiro­[4.5]decane-2,4-dione
Source: Acta Crystallogr E Crystallogr Commun. 2015 Mar 14;71(Pt 4):o238–9. doi: 10.1107/S2056989015004715 (PMC4438798; doi:10.1107/S2056989015004715)

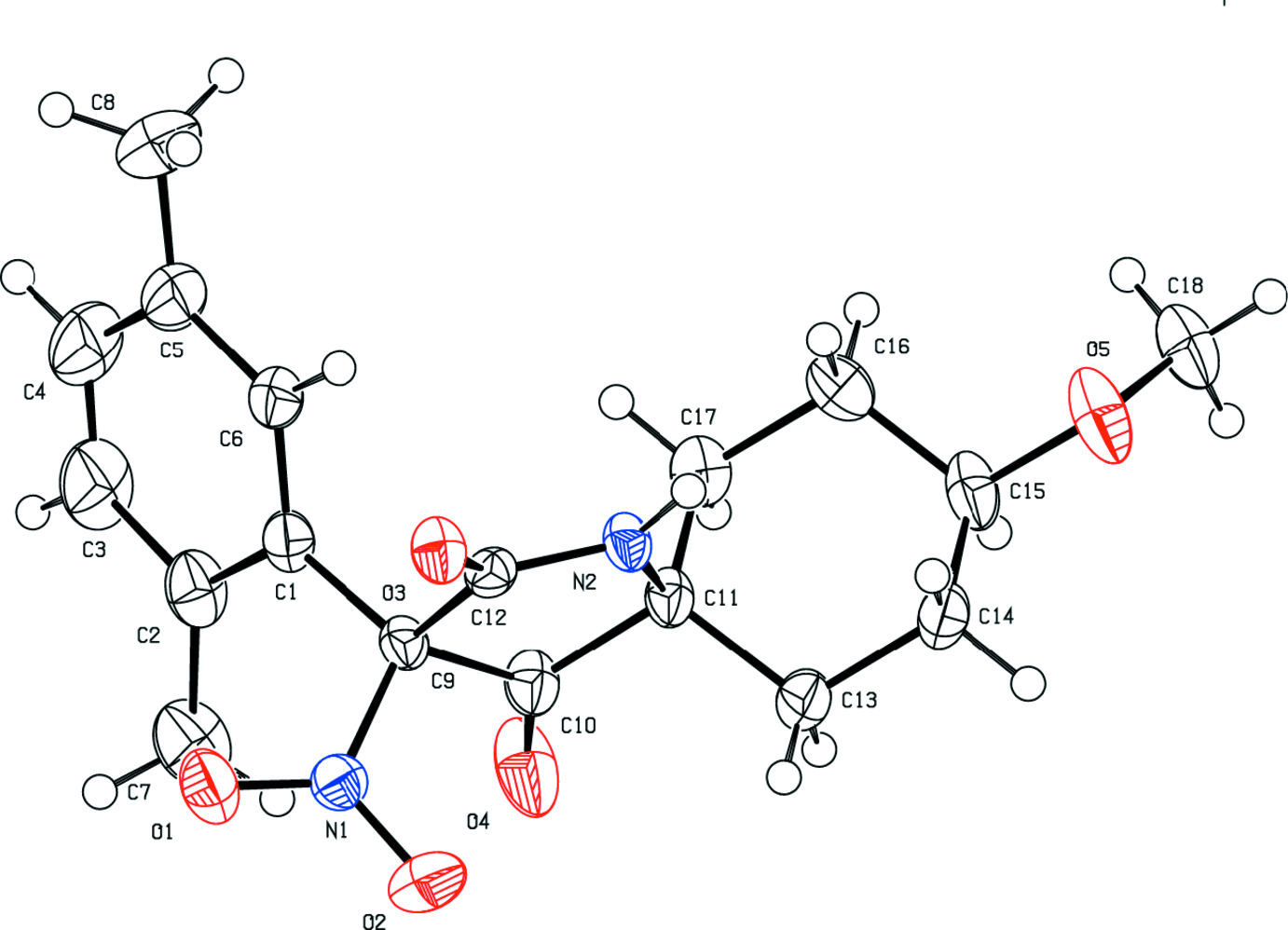

Supplement: Supplementary file 4 [file e-71-0o238-fig1.tif]

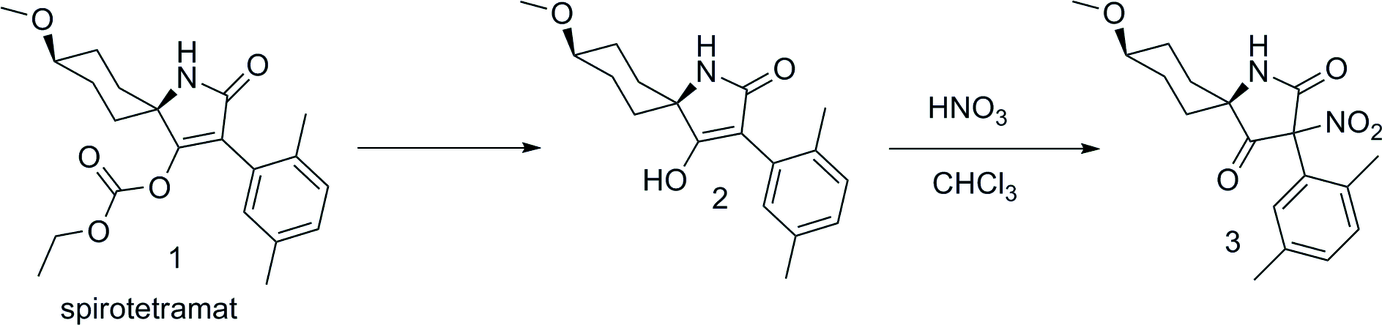

Supplement: Supplementary file 5 [file e-71-0o238-fig2.tif]
